# Supplementary material for: Myristoylated, alanine-rich C-kinase substrate (MARCKS) regulates toll-like receptor 4 signaling in macrophages
Source: Sci Rep. 2023 Nov 10;13:19562. doi: 10.1038/s41598-023-46266-x (PMC10638260; doi:10.1038/s41598-023-46266-x)
Supplement: Supplementary file 1 — Supplementary Information 1. [file 41598_2023_46266_MOESM1_ESM.docx]

# Supplementary Data

# Myristoylated, Alanine-rich C-kinase Substrate (MARCKS) regulates Toll-like receptor 4

# signaling in macrophages.

# Jiraphorn Issara-Amphorn^1^, Virginie Sjoelund^1^, Margery Smelkinson^2^, Sebastian Montalvo^1^, Sung Hwan Yoon^1^, Nathan Paul Manes^2^, Aleksandra Nita-Lazar ^1, #^

# ^1^ Functional Cellular Networks Section, Laboratory of Immune System Biology, National Institute of Allergy and Infectious Diseases, National Institutes of Health, Bethesda, MD 20892-1892, USA

# ^2^ Research Technology Branch, National Institute of Allergy and Infectious Diseases, National Institutes of Health, Bethesda, Maryland 20892, United States.

# ^#^ Corresponding author: nitalazarau@niaid.nih.gov

**Supplementary table 1. Representative MARCKS peptides identified by mass spectrometry.**

| **Sequence** | **Gene names** | Identification WT_IMMs_1 | Identification WT_IMMs_2 | Identification WT_IMMs_2 | Identification  ΔMARCKS_IMMs_1 | Identification  ΔMARCKS_IMMs_2 | Identification  ΔMARCKS_IMMs_3 |
| --- | --- | --- | --- | --- | --- | --- | --- |
| DEAAAAAGGEGAAAPGEQAGGAGAEGAAGGEPR | MARCKS |  |  |  | By MS/MS |  | By MS/MS |
| EAAEAEPAEPSSPAAEAEGASASSTSSPK | MARCKS |  |  |  | By MS/MS | By MS/MS | By matching |
| EAEAAEPEQPEQPEQPAAEEPQAEEQSEAAGEK | MARCKS |  |  |  | By MS/MS | By MS/MS | By MS/MS |
| EELQANGSAPAADKEEPASGSAATPAAAEK | MARCKS |  |  |  | By MS/MS |  |  |
| GEATAERPGEAAVASSPSK | MARCKS |  |  |  | By matching | By matching | By MS/MS |
| LSGFSFK | MARCKS |  |  |  | By matching | By MS/MS | By MS/MS |

**Supplementary table 2. List of primers used in Real-Time PCR experiment.**

| **Name** | **Sequence** |
| --- | --- |
| TNF_F | 5’-CGTCAGCCGATTTGCTATCT-3’ |
| TNF_R | 5’-CGGACTCCGCAAAGTCTAAG-3’ |
| MARCKS_F | 5’-AAGCAAATCGGCACGAGAAT-3’ |
| MARCKS_R | 5’-CCAGCCTCATCCTTTTCG-3’ |

**Supplementary table 3. List of primers used in western blot experiment.**

| **Antigen (origin)** | **Dilutions** | **Company** | **Catalogue Number** |
| --- | --- | --- | --- |
| MARCKS (Mouse) | 1:1000 | Santa Cruz | SC-100777 |
| Beta-actin (Rabbit) | 1:1000 | Cell Signaling Technology | 4970S |
| **Secondary antibodies (origin)** |  |  |  |
| m-IgG Fc BP-HRP (Anti-mouse) | 1:10000 | Santa Cruz | SC-525409 |
| Mouse anti-rabbit IgG-HRP | 1:10000 | Santa Cruz | SC-2357 |

**Supplementary Figure 1.**


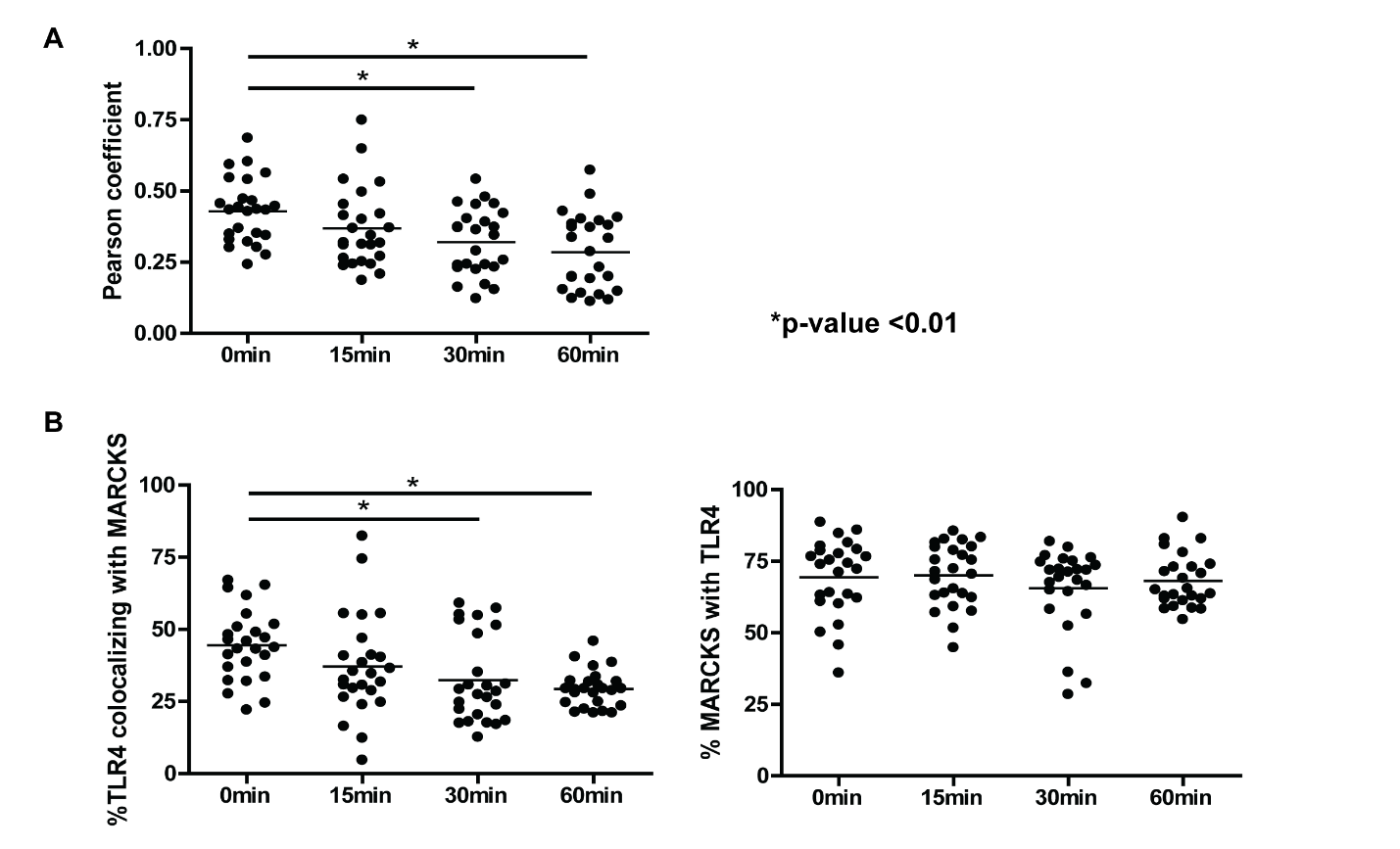


The Pearson coefficient was calculated from the immunofluorescent staining of TLR4 and MARCKS after LPS stimulation (A). The % TLR4 colocalizing with MARCKS and % MARCKS colocalizing with TLR4 (B).

**Supplementary Figure 2. Immunofluorescent staining of MARCKS and TLR4**


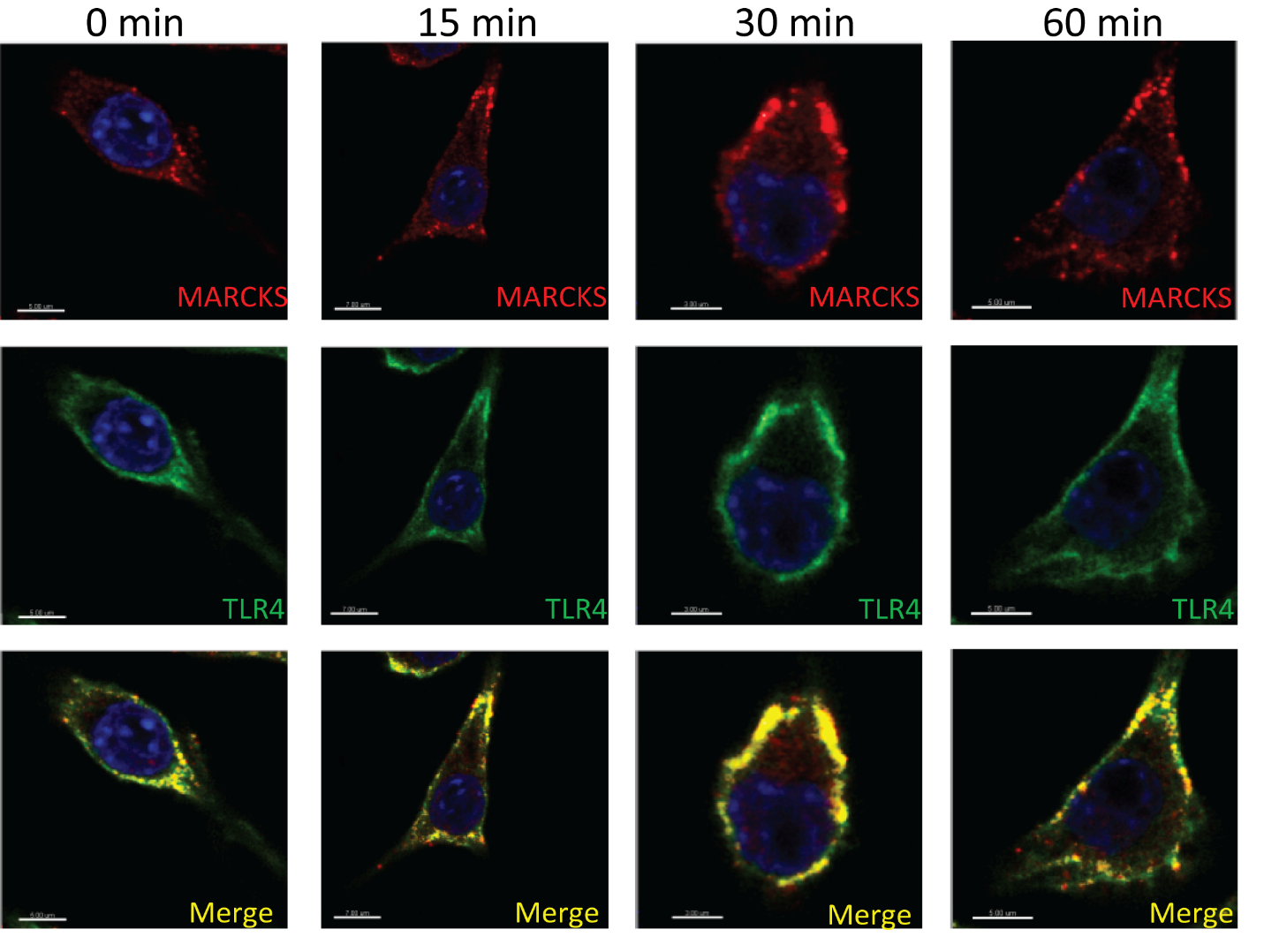


Time course stimulations (0-60 min) of TLR4 (Green) and MARCKS (Red) in macrophages after LPS stimulation. The nuclei were stained with Hoechst (blue). The images of the same samples were merged to show the co-localization of MARCKS and TLR4 (Yellow).

**Supplementary Figure 3. Full-length blots of two independent replicate in this study. Red boxes indicate the areas shown in main figures.**


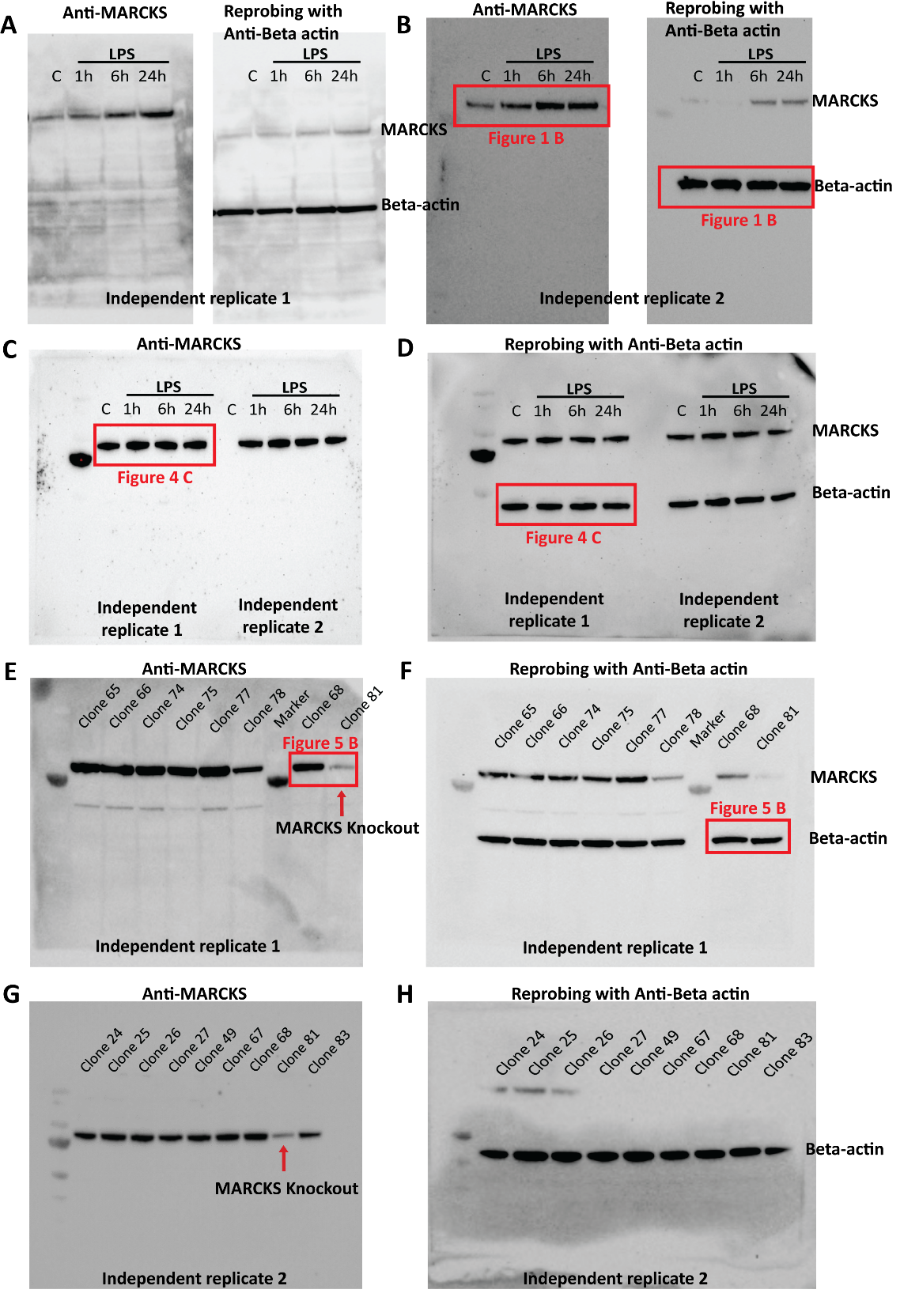


Uncropped full-length images of western blot presented in the main Figure 1B, Figure 4C, Figure 5B. Data are representative of two independent experiments.

**Supplementary Figure 4 The Extracellular Acidification Rate (ECAR)**

WT and ΔMARCKS IMMs were treated with LPS (100 ng/mL) for 6 hours. Extracellular Acidification Rate (ECAR) were measured using Seahorse with indicated inhibitors. The data were normalized to total protein.
